# Vascular reconstruction related to the extracranial vertebral artery: the presentation of the concept and the basis for the establishment of the bypass system

**DOI:** 10.3389/fneur.2023.1202257

**Published:** 2023-06-14

**Authors:** Xuan Wang, Xiaoguang Tong

**Affiliations:** ^1^Department of Neurosurgery, Tianjin Huanhu Hospital, Tianjin, China; ^2^Department of Neurosurgery, Tianjin Central Hospital for Neurosurgery and Neurology, Tianjin, China; ^3^Clinical College of Neurology, Neurosurgery and Neurorehabilitation, Tianjin Medical University, Tianjin, China; ^4^Laboratory of Microneurosurgery, Tianjin Neurosurgical Institute, Tianjin, China; ^5^Tianjin Key Laboratory of Cerebral Vascular and Neural Degenerative Diseases, Tianjin, China

**Keywords:** extracranial vertebral artery, cerebral revascularization, posterior circulation bypass, high-flow bypass, vascular reconstruction system

## Abstract

The intracranial vertebrobasilar artery system has a unique hemodynamic pattern (vessel trunk converged bilateral flow with three groups of perforators directly arising from it), is embedded within intense osseous constraints, and is located far from conventional donor vessels. Two major traditional modalities of posterior circulation revascularization encompass the superficial temporal artery to the superior cerebellar artery and the occipital artery to the posteroinferior cerebellar artery anastomosis, which are extracranial-intracranial low-flow bypass with donor arteries belonging to the anterior circulation and mainly supply focal perforators and distal vascular territories. As our understanding of flow hemodynamics has improved, the extracranial vertebral artery-related bypass has further evolved to improve the cerebral revascularization system. In this article, we propose the concept of “vascular reconstruction related to the extracranial vertebral artery” and review the design philosophy of the available innovative modalities in the respective segments. V1 transposition overcomes the issue of high rates of in-stent restenosis and provides a durable complementary alternative to endovascular treatment. V2 bypass serves as an extracranial communication pathway between the anterior and posterior circulation, providing the advantages of high-flow, short interposition grafts, orthograde flow in the vertebrobasilar system, and avoiding complex skull base manipulation. V3 bypass is characterized by profound and simultaneous vascular reconstruction of the posterior circulation, which is achieved by intracranial-intracranial or multiple bypasses in conjunction with skull base techniques. These posterior circulation vessels not only play a pivotal role in the bypass modalities designed for vertebrobasilar lesions but can also be implemented to revascularize the anterior circulation, thereby becoming a systematic methodology.

## 1. Introduction

There is great potential for perfection and continual improvement of the posterior circulation bypass system, which poses obvious contrasts to its anterior circulation counterpart with formulated patterns. The revascularization of the brainstem makes these procedures a formidable challenge since the intracranial vertebrobasilar artery system is confined by intense osseous constraints and is located far from conventional donor vessels. In our opinion, the underlying reason lies in the unique hemodynamic pattern of the posterior circulation, which is distinct from the simple structure of the anterior circulation with a single group of perforators located proximal to the vascular tree. The vessel trunk has converged bilateral flow with three groups of perforators directly arising from it, including the basilar apex, basilar trunk, and vertebral artery (VA) adjacent to the posteroinferior cerebellar artery (PICA). The Ausman team pioneered the use of the superior temporal artery (STA) to bypass either the superior cerebellar artery (SCA) or the posterior cerebral artery (PCA) and the occipital artery (OA) during PICA bypass procedures. They used these arteries to create tunnels that redirected blood flow to the vertebrobasilar territories through recipient transfer, which formed the foundation of the modern posterior circulation bypass system (1, 2). These two classic modalities are extracranial-intracranial low-flow bypass with donor arteries from the anterior circulation and mainly focus on their respective focal perforators. As the understanding of flow hemodynamics, skull base techniques, and cervical anatomy has improved, novel configurations for cerebral reconstruction have emerged, such as short graft medium-flow bypass supplied by the internal maxillary artery (IMA) and the extracranial vertebral artery-related bypass (3), which further revolutionized the cerebral revascularization system. However, their benefits are not yet apparent and require further investigation.

**Figure 1:**
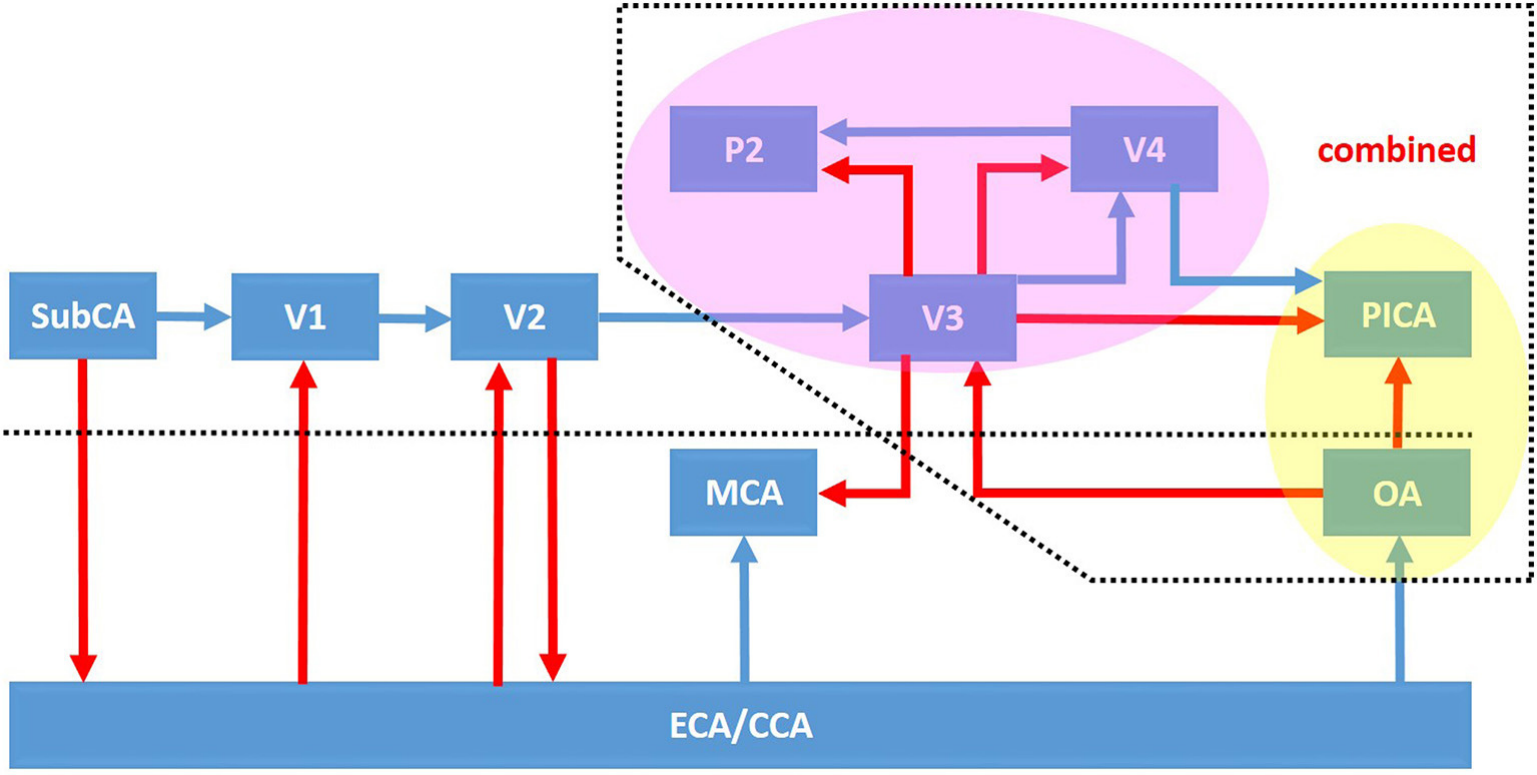
Schematic illustration of vascular reconstruction related to the extracranial vertebral artery of the respective segments. The bypass flow (red arrows) and original flow (blue arrows) are indicated in the pictures. The bypass configurations labeled in the pink and yellow areas could be established in combination for the vascular reconstruction of the overall posterior circulation. CCA, common carotid artery; ECA, external carotid artery; MCA, middle cerebral artery; OA, occipital artery; PICA, posterior inferior cerebellar artery; P2, P2 segment of the posterior cerebral artery; SubCA, subclavian artery.

**Figure 2:**
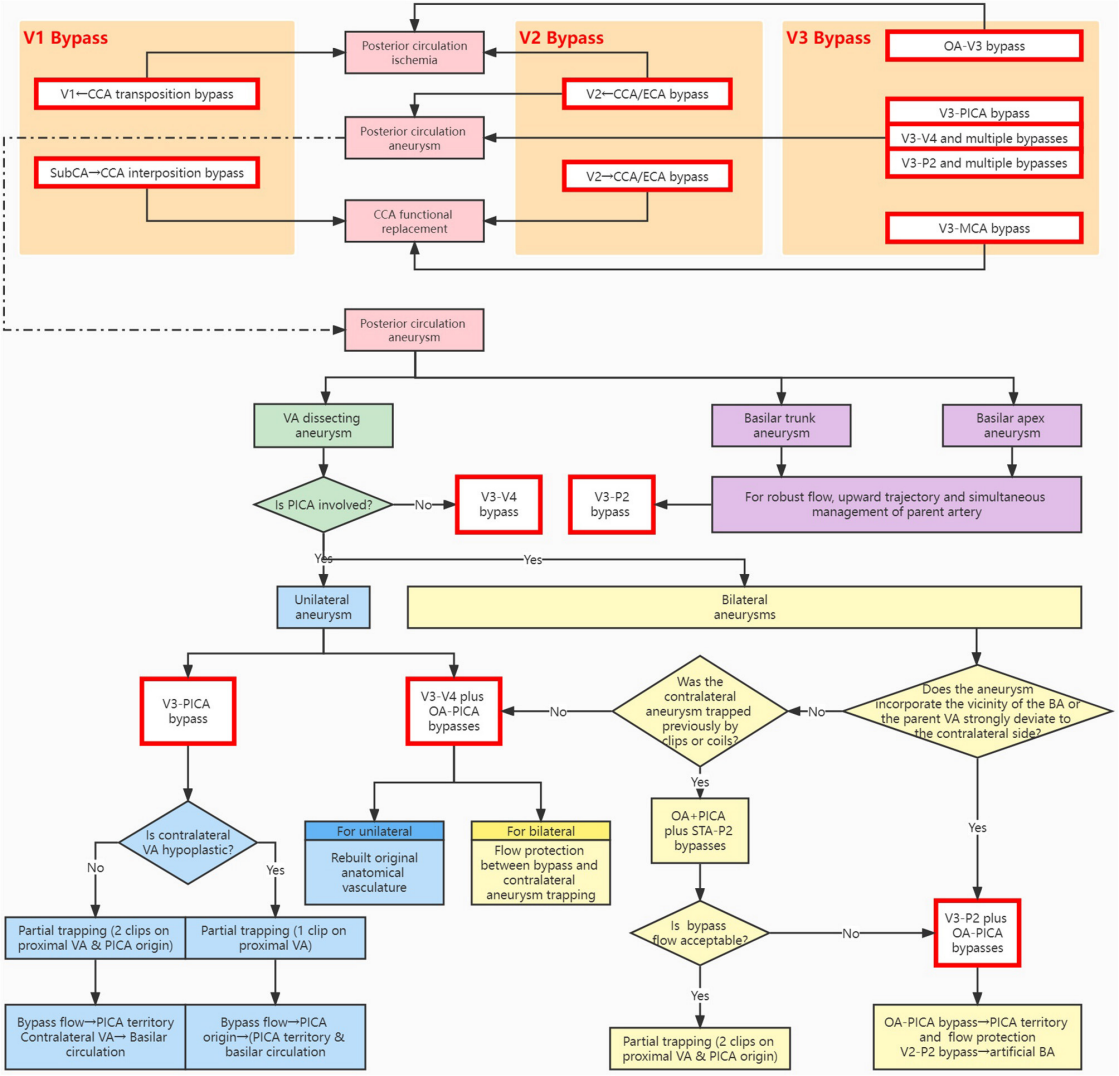
Schematic algorithm for bypass strategy of the vascular reconstruction system related to the extracranial vertebral artery. The main indications include posterior circulation ischemia, CCA functional replacement, and posterior circulation aneurysms. The methodological approach illustrates the design philosophy of the available modalities in the respective segments. BA, basilar artery; CCA, common carotid artery; ECA, external carotid artery; MCA, middle cerebral artery; OA, occipital artery; PICA, posterior inferior cerebellar artery; P2, P2 segment of the posterior cerebral artery; STA, superficial temporal artery; SubCA, subclavian artery; VA, vertebral artery; V1/V2/V3/V4, V1/V2/V3/V4 segment of the vertebral artery.

The VA is divided into four segments: the V1–V3 extracranial and the V4 intracranial. The V1 segment originates from the subclavian artery (SubCA) and extends to its point of entry into the transverse foramen of the C6 vertebra; the V2 segment courses within the transverse canal (4, 5), and the V3 segment extends from the C1 transverse foramen to the point at which the VA enters the dura (V3 can also be defined as the tortuous atlantal portion of the VA distal to the C2 transverse foramen) (6, 7). Although the anterolateral cervical approach allows the exposure of the whole length of the extracranial VA through the dissecting space between the sternocleidomastoid muscle (SCM) and the internal jugular vein (IJV) (7), yielding safe and direct visualization of the VA, previous bypass procedures were specifically applied depending on the associated pathologies of the different segments. Therefore, the title of the related chapter in Youmans' neurological surgery was still “*Extracranial vertebral artery diseases*” (4). The development of multiple bypass modalities represents an important conversion of the extracranial VA from an object to a subject of treatment. These posterior circulation vessels not only play a pivotal role in the bypass modality designed for vertebrobasilar lesions but can also be implemented to revascularize the anterior circulation, becoming a systematic methodology comprised of cervical bypass, communicating bypass, extracranial-extracranial bypass, intracranial-intracranial bypass, skull base bypass, and multiple bypasses, which differ from the specific modified procedures such as the OA-SCA and PICA-PICA bypass. Therefore, we propose the concept of “vascular reconstruction related to the extradural vertebral artery” and reviewed the design philosophy of the available innovative modalities in the respective segments (Figures 1, 2).

## 2. VA/V1 segment bypass

### 2.1. Revascularization procedures applied for the V1 segment

Proximal VA revascularization is mainly indicated for stenosis or occlusion of the VA orifice (4–8) via VA endarterectomy or transposition (9). Unlike the proximal ICA, the small caliber and deep location of the V1 orifice near the thoracic inlet make exposure challenging (4), and pure VA endarterectomy is seldom used (9). In comparison, transposition of the proximal VA onto the CCA is the most common and safe procedure (Figure 1) (10), and the V1 lacking branch vessels is more feasible to separate and mobilize than the V2 transversary segment for reimplantation into the neighboring vessel (4). Other available options for implantation sites include the SubCA or thyrocervical trunk with or without an interposition graft (4). The surgical field for the V1 region is situated between the anterior scalene muscle and the carotid sheath in close proximity to multiple complex and important structures. Manipulation of this region is associated with severe complications, including Horner's syndrome, chylothorax, and recurrent laryngeal nerve paralysis (4, 10). With the advent of endovascular techniques, balloon angioplasty, and stent implantation have become popular due to their gentle learning curve, minimally invasive nature, and shorter anesthetic duration compared to open surgery (11).

However, several recent studies have revealed the high rates of in-stent restenosis (ISR) of the VA orifice, which led to questions concerning the effectiveness of VA stenting. The possible cause remains unclear and may be associated with the well-developed muscular layer of the VA orifice (11, 12). The SSYLVIA trial revealed that the postoperative (6 months later) ISR rate of the extracranial VA stent accounted for up to 42.9% (6/14) of the cases, of which 66.7% (4/6) occurred at the VA orifice (13). Despite the small number of cases, microsurgical revascularization has recently attracted great attention (9–11). VA transposition appears to be more functional in inhibiting recurrent stenosis than VA endarterectomy (9). Rangel-Castilla et al. reported that the restenosis rate of VA-CCA transposition was 4.5% (1/22) at the average 8.8-month follow-up (10). Berguer and colleagues presented a series that enrolled 230 patients treated by microsurgical revascularization of the proximal VA. The patency rate was more than 90% at 10 years, despite the inherent risks of postoperative complications. Few patients experienced ischemic events (1.9%), and the overall remission rate of complications was 83% (10, 14).

### 2.2. Surgical approach and protection of the adjacent structures

The standard method for proximal VA exposure is the supraclavicular approach, which focuses on the VA origin in the SubCA. The supraclavicular incision parallels the clavicle, and the clavicular head of the SCM is cut and retracted upward (4, 11). In this study, the vessel was identified medially to the thyrocervical trunk as a landmark with a dissection plane between the CCA medially and the IJV laterally. This route was applied in pure VA endarterectomy to achieve extensive exposure of the SubCA for temporal occlusion of the thyrocervical trunk and internal thoracic artery and to facilitate concomitant subclavian endarterectomy for plaques extended to the SubCA (4, 9). Similarly, carrying out the bypass procedure using interposition grafts from the SubCA or the thyrocervical trunk could also be beneficial for this approach.

However, direct exposure to the supraclavicular approach requires in-depth knowledge of relevant low-lying anatomy unfamiliar to neurosurgeons. George et al. preferred primary exposure of the distal V1 segment at the C6 transverse foramen (the most caudal transverse process could be palpated) and then safely traced proximal to the VA origin through the field between the SCM and the IJV (9, 15). The skin incision extends along the medial border of the inferior part of the medial border of the SCM, which is retracted laterally without division. This approach is an optimal choice for VA-CCA transposition. It shares major steps with the anterolateral cervical approach to the V2 segment coursed in the transverse canal and does not pursue exposure with more inferior extension toward the SubCA (7, 15). The numerous adjacent important structures are mainly contained within the “VA triangle.” This relationship creates a practical map to ensure prompt recognition and avoid iatrogenic injury (16). This concept was introduced by Tubbs et al. to describe the muscular bed between the retrojugular fat pad and the VA, where the longus colli and the anterior scalene muscles converge at the C6 transverse process and outline the triangle with the BA as its base. The omohyoid muscle crosses the VA triangle and can be divided for wider exposure (4, 16). The vagus nerve runs beneath the IJV (17) and does not enter the VA triangle; it is usually mobilized along with the IJV, either lateralized to expose the SubCA in the anterolateral cervical approach or medialized for a key step of the supraclavian approach to find the retrojugular fat pad that overlies the VA triangle (16). It is worth noting that vocal cord paralysis may result from excessive retraction since the right recurrent laryngeal nerve exits this nerve and winds around the SubCA (higher up than the nerve loops below the aortic arch on the left side) (4). The supraclavian and anterolateral approaches allow different routes of access through the lower or upper half of the VA triangle, respectively. During the supraclavian procedure, lateral dissection should not proceed beyond the anterior scalene muscle to avoid damage to the brachial plexus located laterally in the scalene space and the phrenic nerve lying on the surface of this muscle.

Similarly, the sympathetic chain does not run strictly along the medial muscular border of the VA triangle; it enters the VA triangle inferiorly to pass through the stellate ganglion and cross the proximal VA. Meticulous care must be taken when dissection from the C6 transverse process follows the longus colli muscle to prevent the occurrence of Horner's syndrome (15, 16). George et al. recommended the use of the aponeurosis of the longus colli muscle rolled around the sympathetic chain for protection (15). In addition to the sympathetic chain, two important structures cross the V1 segment, which is located within the lower portion of the VA triangle. The lymphatic vessel (the thoracic duct on the left side) accompanies the SubCA near the V1 origin. The inferior thyroid artery branches from the thyrocervical trunk and blocks the superior portion of the V1 segment (15, 16). Both of them could be ligated and separated if necessary to avoid injury to the thoracic duct, which could result in chylothorax (9).

### 2.3. Extended application of the V1 region bypass

The procedure for the exposure of the V1 region involves another valuable application in supplying high flow from the SubCA for the treatment of CCA occlusion ischemia. Symptomatic occlusion of the CCA constitutes 2–4% of all cases of carotid circulation occlusion, and SubCA to carotid bypass is particularly effective for long-segment occlusion (Figures 1, 2) (18). The donor and recipient vessels are exposed through two separate incisions: the supraclavicular and pre-SCM trajectories for the SubCA and the carotid artery, respectively. The recipient site varies from the distal CCA to the proximal ICA, depending on the occlusion level, with the polytetrafluoroethylene (PTFE) conduit (not prone to kinking like the SVG) tunneled behind the IJV and ventral to the vagus nerve and phrenic nerve (18). Due to the appropriate length of the interposition graft, Illuminati et al. also employed this technique for the aggressive en bloc resection of recurrent cervical malignant tumors, and it allowed for the simultaneous replacement of the affected CCA (19). Another revascularization option involved introducing flow from the contralateral carotid artery via the retropharyngeal route; however, the short graft length does not justify this treatment in patients who cannot tolerate temporal occlusion of the remaining patent CCA. Unless the main branches of the aortic arch are unavailable, this alternative is preferred for the CCA occlusion if the surgeon is familiar with the complex anatomy of this region (see below for the V2 bypass P → A type and V3-MCA modality) (18).

## 3. VA/V2 segment bypass

### 3.1. The posterior communicating artery Bypass

The early focus of V2 segment-related treatment for posterior circulation ischemia was limited to the decompression of osteophytic VA stenosis at the transverse foramen (20, 21). In addition, Gerke et al. reported that a V2 traumatic aneurysm occurred with distal stenosis of the parent artery, which was obliterated. The ECA was incised and directly anastomosed to the VA at the C2–C3 level (22). Along with the expansion of the application field, the V2 bypass contributed to the improvement of the posterior circulation bypass system. Since the V2 bypass connected the extracranial VA with the cervical carotid artery, which lies in close proximity, it constructed a pivotal bridge that directly communicates the anterior and posterior circulation and functions as an extracranial PCOM (Figure 1) (23).

### 3.2. V2 bypass conducting flow from the anterior to the posterior circulation (A → P type)

This typical approach is primarily indicated for posterior circulation ischemia (Figure 2). Carney and Anderson initiated the V2 bypass in a symptomatic carotid occlusion patient. The parietal and occipital lobe infarcts that occurred due to the internal steal effect for flow were diverted away from the vertebrobasilar to the carotid territories through the PCOM. The large-caliber recipient VA of V2 bypassed for flow augmentation allowed for simultaneous improvement of anterior and posterior circulation perfusion and constituted a viable alternative superior to the STA-MCA bypass (24). Considering the advantages of its high flow capacity and the orthograde direction of vertebrobasilar cannulation, the V2 bypass was straightforwardly used for vertebrobasilar ischemia. The V2 bypass was introduced by Camp et al. and has already become a standard operation (22, 25–27). However, it is essential for the collateral supply from the OA muscular branch or ascending cervical artery to reconstitute a sufficient length of the distal cervical VA (28), which serves as part of the common pathway of this bypass configuration (22, 23, 26, 27).

### 3.3. V2 bypass conducting flow from the posterior to the anterior circulation (P → A type)

The bypass flow is seldom oriented in this infrequent direction, and its merit lies in the revascularization of long-segment CCA lesions, but its main impediment is the lack of an appropriate high-flow donor source (Figure 2). This idea originated from the management of Takayasu's arteritis with a bypass procedure. Ziyal et al. selected the V3 segment to substitute the aortic arch as a donor, forming a key part of the overall replacement of the aortic arch and its major branches (29). Li et al. were the first to switch this novel donor site to V2 for CCA occlusion caused by Marfan syndrome to avoid the “jump bypass” between the remotely situated recipient vessel and V3 in far-lateral exposure (30), as well as its potential damage to the collateral branches from SubCA or OA, which retrogradely reperfuses the carotid artery. For the remaining available options for CCA revascularization, the “Bonnet” bypass utilized the contralateral STA or ECA as the donor source and required an interposed graft crossing the dome of the calvarium to the MCA, significantly increasing the length of the graft and posing the risk of mechanical damage (31). The V3 bypass is more applicable for directly supplying the intracranial territories when the cervical carotid luminal is infeasible as a flow pathway (see below for the V3-MCA modality).

### 3.4. Hybrid operation

In addition to the role of the flow modulation pathway, the V2 bypass establishes a physical trans-circulation route for endovascular therapy (Figure 2). Chwajol et al. adopted this extracranial “PCOM” for endovascular access to reach intracranial complex posterior circulation aneurysms when tortuous or proximal occluded VA prevented routine catheterization, and the true PCOM was often hypoplastic to deliver the endovascular materials in the meantime. It is a valuable method to overcome the difficulty of releasing multiple loops of kinks in which transverse foramen unroofing or reroute techniques fail to restore endovascular access. Additionally, direct surgical exposure of the cervical VA to circumvent the prohibitive vessel anatomy for endovascular catheterization requires only a single operation, whereas the V2 bypass is convenient for potential subsequent endovascular treatments (32).

## 4. VA/V3 segment bypass

### 4.1. Overview and exposure of the V3 and OA

The general trend of the V1 bypass to the V3 bypass is that the role played by the extracranial VA is shifted from the recipient to the donor. A situation in which the V1 segment received blood flow and was transformed to V2 bypass served as a communication pathway, and V3 appeared to be a notable source due to its robustness and tolerance to temporary occlusion (with contralateral VA still supplying the basilar territory) (Figure 1) (33). Although V3 is located deep within the suboccipital triangle, it can be accessed from the intracranial surgical field of diverse recipient arteries without a second remote site and is considered a representative donor of IC-IC as well as IMA (33). The characteristic application for V3 bypass is profound and requires simultaneous vascular reconstruction of the entire posterior circulation (every component, including the PCA/SCA, BA, VA, PICA, etc.) in conjunction with advancing skull base techniques. Localizing the VA and minimizing the blood loss of the accompanying veins are the key points for exposing the V3. Tayebi Meybodi et al. (34) proposed the atlanto-mastoid line (which runs between the mastoid tip and C1 posterior tubercle) or the belly of the superior oblique capitis muscle to guide the exposure of V3. Wanibuchi et al. (35) presented a systematic method using bony landmarks to identify the “J-groove” on the posterior arch of C1, which cradles the VA. When the extracranial VA between the C1 and C2 vertebrae is used to replace V3 in the reconstructive procedure, the subatlantic triangle (formed by the levator scapulae muscle and the splenius cervicis muscle inferiorly and laterally, the longissimus capitis muscle inferiorly and medially, and the inferior oblique capitis superiorly), which is located inferolateral to the suboccipital triangle, offers a direct gateway to expose this vessel (36). Arnautović et al. named the surrounding venous compartment (not the venous plexus) of V3 the “suboccipital cavernous sinus” due to its analogy to the cavernous sinus that cushions the ICA (37). This venous compartment is covered by the posterior atlantooccipital membrane and is separated from the overlying deep suboccipital muscle. Youssef et al. (38) applied the interfascial dissection technique to dissect the natural tissue plane between the membrane and the muscle fascia in a blunt fashion to achieve bloodless exposure of the V3.

The OA is another major donor for posterior circulation and is particularly important in V3 bypass, which could be modified or combined with the traditional OA-PICA bypass. The Japanese authors advocate multiple-layer dissection of the suboccipital muscles to accomplish a far-lateral approach for harvesting the OA, which is ~15 cm in length (prior to OA harvest, the SCM has been reflected anteriorly, the splenius capitis muscle posteroinferior, the longissimus capitis muscle inferiorly, then the semispinalis capitis muscle is reflected posteriorly, the superior oblique capitis muscle anteriorly, the rectus capitis posteriorly, and the major muscle posteriorly). This procedure helps with the exposure of V3 in the suboccipital triangle and the creation of a shallower surgical field (39, 40). Fukuda et al. (40) proposed that understanding the concept of “the transitional segment of the OA” is crucial for simplifying the harvesting procedure. The transitional segment extends from the superior edge of the splenius capitis muscle to the superior nuchal line, located where the OA pierces two anatomical planes (the tendon of the SCM and the galea aponeurotica) vertically. The corresponding reverse-C skin incision [both ends on the middle lines located on the external occipital protuberance and the spinous of C2, the apex on the mastoid process, a variant of Rhoton's inverted horseshoe incision (with a longer lateral limb) (41)] is beneficial to first expose the SCM (the lateral edge) and the splenius capitis muscle (the superior border) as landmarks to isolate the transitional segment, followed by dissecting the intramuscular segment proximally and subcutaneous segment distally within the single tissue layer (the styloid diaphragm and the epigaleal layer, respectively) (40).

### 4.2. OA-V3 modality

This bypass (Figures 1, 2) was initiated by the Spetzler team in a case of traumatic pseudoaneurysm of the cervical VA for flow replacement after aneurysm trapping (20, 42). Wang et al. first developed this bypass modality as a novel treatment option for bilateral vertebral steno-occlusive disease (28). OA-V3 bypass offers several major advantages over conventional OA-PICA bypass, including making the surgical field shallower and wider to avoid deep anastomosis, sparing patients invasive craniotomy and intracranial manipulation, decreasing the operative time and anesthetic duration, and broadening the application scope of posterior circulation bypass for ischemia (28). The potential limitations of the OA-V3 bypass are a caliber discrepancy between the donor and recipient vessels, rigorous criteria restrictions for selecting patients with hemodynamic compromise, and the various anastomotic techniques (e.g., double barrel bypass) employed to ensure the patency of the bypass and that lead to orthograde filling of the upper posterior circulation (28). The V2 bypass might seem attractive to surgeons who are not familiar with the dissection of the suboccipital musculature. However, this alternative bypass strategy requires an interposition graft and two anastomoses and is more suitable for VA occlusion that does not extensively involve the V2.

### 4.3. V3-PICA modality

This strategy is another alternative for OA-PICA bypass rather than the OA-V3 modality (Figures 1, 2), which was developed for the treatment of VA dissecting aneurysms encompassing the PICA origin with the evolution of the donor's vessel from V4 to V3. Durward initially implanted the PICA origin proximal to the vessel dissection to maintain its normal blood flow after aneurysm trapping (43). This bypass option eliminates the tedious dissection required to harvest the OA and the potential risk of hypoperfusion due to the diminutive caliber of the OA, providing an excellent substitute for OA-PICA (43, 44). However, this procedure is technically sophisticated, and the perforators of the medulla oblongata that arise from the proximal PICA may be damaged. Very few cases have enough PICA redundancy to allow for tension-free anastomosis; thus, Hamada et al. employed the STA as an interposition graft to accomplish PICA reimplantation (45). Benes et al. optimized the deep surgical space by using transcondylar and transjugular tubercle approaches, helping to simplify the direct anastomosis between the V4 and PICA without starting with the graft (46).

It should not be ignored that we cannot ensure that the reimplantation site on V4 completely avoids the pathological vessel wall. Hence, Czabanka et al. altered the proximal donor source to V3 with a radial artery graft to match the flow demand of the PICA (44). Similar to PICA reimplantation, the V3-PICA bypass revascularizes the PICA territory, while the basilar circulation is supplied by the contralateral VA (44, 47). Beyond this routine indication, in situations where the contralateral VA is hypoplastic, proximal inflow occlusion of the parent artery is performed without placing the clamp on the PICA origin, which produces a retrograde flow of the V3-PICA bypass to supply the entire basilar territory. When a conventional OA-PICA bypass is confirmed to be occluded intraoperatively, the OA could be transected proximally and mobilized to the V3 segment; here, the V3-PICA bypass was used to re-establish antegrade perfusion as a salvage maneuver (44).

### 4.4. V3–V4 modality and related multiple bypasses

This bypass was originally designed for local lesions as well as the OA-V3 bypass. In a case of a giant partial thrombosed VA aneurysm distal to the PICA origin where clip reconstruction failed, Evans et al. employed an SVG insert between the extracranial and intracranial VAs following aneurysm excision via a transsigmoid approach (48), similar to the creation of an interposition graft to bridge the gap in the parent artery for MCA aneurysms. Shi et al. even resected and replaced bilateral VA aneurysms located proximal to the PICA with RA grafts (49). This replacement strategy also highlights its importance in the treatment of complex skull base tumors with extensive involvement, with no requirement for deciding between radical resection of the tumor and preservation of the encased or invaded VA (50, 51). Once the VA lesion affects the PICA, the V3–V4 bypass could be advanced by integrating the OA-PICA bypass to reconstruct the entire posterior circulation (Figures 1, 2).

In retrospect, the prototype of these multiple bypasses originated from a failed endovascular angioplasty case of symptomatic stenosis at the entry segment of the VA. The surgical procedure was altered to a V3–V4 replacement bypass. To protect the PICA and BA territories during temporal occlusion due to contralateral VA occlusion, the OA was anastomosed to the contralateral PICA, which was dominant and perfused from the operative side VA (52). The characteristic configuration of multiple bypasses was established to address bilateral VA fusiform aneurysms and reconstruct the intracranial VA and ipsilateral PICA prior to aneurysm trapping. Moreover, the prolonged period of temporary flow arrest required for multiple anastomoses was compensated for with the contralateral VA, and the opposite aneurysm could be obliterated by the endovascular occlusion of the parent artery in the second stage, with the bypass flow serving as collateral circulation in turn (53) (Figure 2). Although less complicated, the V3-PICA bypass is unsuitable in cases without a favorable contralateral VA (44). Instead, V3–V4 coupled with OA-PICA bypass can restore the original anatomical vasculature and can even be used for unilateral VA aneurysms by experienced surgeons (54, 55). If the OA-PICA bypass is occluded intraoperatively because of thromboembolism, the reimplantation of the proximal OA to the interposition graft of the V3–V4 bypass can be employed for rescue adjustment (55). For large perforators emanating from the aneurysm dome, it is possible to perform OA-perforator bypass to maintain the flow to the brainstem-perforator vessels, similar to the PICA territory during VA reconstruction. However, the indications for perforator bypass are yet undefined (56).

### 4.5. V3-P2 modality and related multiple bypasses

The V3-P2 bypass takes advantage of the upward trajectory of the posterior petrosal approach. The ideal perpendicular course allows P2 to be directly approached without excessive retraction of the temporal lobe (57), and a similar principle is applicable for retrochiasmatic craniopharyngiomas (58), which is especially suitable for giant basilar apex aneurysms (59) and provides excellent access to high-positioned basilar quadrification that is distorted by dolichoectatic basilar trunk aneurysms (60). The reliable collateral flow established by the bypass ensures terminal BA occlusion to eliminate the flow jet effect. This flow diversion converts the basilar apex aneurysm into a “sidewall” aneurysm, facilitating aneurysm involution (59). The successful treatment of basilar trunk fusiform aneurysms (including the dolichoectatic type) lies in robust retrograde flow to alleviate the development of vascular dissection and intramural thrombus deposition (60, 61). The flow capacity of the V3-P2 bypass meets the above requirements and ensures adequate perfusion of the brainstem perforators of the basilar trunk (Figure 2).

Horie et al. (62) reported a patient with a basilar trunk giant thrombosed fusiform aneurysm who underwent STA-P2 bypass first but resorted to V3-P2 bypass when BTO failure indicated insufficient collateral reserve of the conventional donor. Although this bypass is amenable to simultaneous management of the lower BA and ipsilateral VA, such as parent artery occlusion or trapping, expertise in skull base surgery is required. Mai et al. integrated the surgical fields of the posterior transpetrosal and far lateral approaches to accommodate the interposition graft, but the sophisticated operation may be applied as a 2-day procedure (59). The bone work may be simplified to a combination of subtemporal and far-lateral craniotomies, albeit at the expense of a redundant graft length, which spans between the supratentorial and infratentorial exposure areas for the graft vessel and needs to detour around the mastoid bone (60).

V3-P2-related multiple bypasses were originally introduced as a viable alternative to their V3–V4 counterparts for bilateral VA dissecting aneurysms (Figure 2). When the sacrifice of the unilateral VA has the risk of opposite aneurysm enlargement, owing to hemodynamic stress, the V3–V4 bypass cannot depend on the contralateral parent artery to provide collateral flow during the occlusion time. For this purpose, Saito et al. adopted the STA-SCA plus OA-PICA bypass (via a combined petrosal approach) to revascularize the upper and lower halves of the posterior circulation, respectively, with the V3-P2 bypass planned as a standby, which was finally avoided due to the neurophysiological parameters and perfusion pressure indicating an acceptable bypass flow (63). In addition, if the aneurysms incorporate the vicinity of the BA or the parent VA strongly deviates to the contralateral side, the recipient site of the V4 segment is not feasible for V3–V4 bypass; thus, V3-P2-related multiple bypasses could be prepared.

This reconstruction configuration is so technically challenging among the V3 bypass options that only Ota et al. reported one case accomplished through a transcondylar fossa approach along with presigmoid exposure, where the OA-PICA bypass revascularized the affected PICA and worked in collaboration with the STA-SCA bypass to protect flow during the anastomosis. The high-flow V3-P2 bypass rebuilt an artificial BA morphologically, and the distal anastomosis between the graft and P2 was dispensed with temporary occlusion of the ispilateral VA and achieved a shorter duration of potential ischemia, despite the filling pattern of the posterior circulation shifting to flow reversal (64). Kubota et al. proposed that the ECA be connected to V4 (or the lower BA) when the V3 segment was not available as the donor site; here, the STA-SCA coupled with the OA-AICA maintained collateral flow as well (55).

### 4.6. V3-MCA modality

The V3 segment also allows revascularization of the anterior circulation other than the vertebrobasilar system, which is especially suitable for CCA occlusion and presents a challenge due to the absence of conventional donor vessels such as the STA (Figure 2). Schneider et al. (65) established an anastomotic connection between the V3 and M2 segments of the MCA to restore the flow of the anterior circulation compromised by CCA occlusion, albeit without the intrinsic properties of authentic “PCOM” bypass, such as a short graft and bidirectional flow modulation. This bypass curtailed the graft length to an acceptable limit compared to the “bonnet bypass” and provided more robust blood flow than the contralateral donor STA. For both feasible options, utilizing the SubCA to reconstruct the CCA (18) or employing V2 cervical PCOM bypass (30), only selected cases met the demands that the residual lumen of the ICA be reconstituted as a recipient conduit. In contrast, the V3 segment is likely to be widely used due to its direct filling of the objective MCA territory (65) and its considerable high flow (peak flows of more than 70 ml/min, with average flows in the range of 25 ml/min) for effective flow replacement (66).

Miele et al. reported a patient with a giant supraclinoid ICA aneurysm who had undergone previous Hunterian ligation of the CCA. The initial STA-MCA bypass (ECA and ICA partially compensated by the OA muscular branch) failed to improve the perfusion. The coiled aneurysm was recanalized due to the continuous supply of the PCOM. Under the security of the second V3-MCA high-flow bypass, an endovascular sacrifice of the feeding PCOM was tolerated to achieve complete aneurysm obliteration, which is not appropriate for V2 bypass because of recipient pathway occlusion by aneurysm trapping (66). This bypass configuration has multiple applications to address cervical tumors. A replacement procedure is indicated when the malignant tumor infiltrates the carotid artery during radical tumor resection or is occluded by local radiation therapy, both of which frequently occur (33). Specifically, in a case with an infected salivary fistula followed by wide neck dissection, V3-MCA bypass helps reroute the interposition graft to circumvent the infected surgical field and avoid graft complications (67).

## Author contributions

XW and XT: conception and design. XW: drafting of the article. XT: critical revision of the article and study supervision. All authors contributed to the article and approved the submitted version.
